# A Mathematical Model of *Campylobacter* Dynamics Within a Broiler Flock

**DOI:** 10.3389/fmicb.2019.01940

**Published:** 2019-08-21

**Authors:** Thomas Rawson, Marian Stamp Dawkins, Michael B. Bonsall

**Affiliations:** ^1^Mathematical Ecology Research Group, Department of Zoology, University of Oxford, Oxford, United Kingdom; ^2^Department of Zoology, University of Oxford, John Krebs Field Station, Oxford, United Kingdom

**Keywords:** *Campylobacter*, mathematical modeling, stochastic differential equations, bacterial population dynamics, microbiome

## Abstract

Globally, the bacterial genus *Campylobacter* is one of the leading causes of human gastroenteritis, with its primary route of infection being through poultry meat. The application of biosecurity measures is currently limited by a lack of understanding of the transmission dynamics within a flock. Our work is the first to undertake a mathematical modeling approach to *Campylobacter* population dynamics within a flock of broilers (chickens bred specifically for meat). A system of stochastic differential equations is used to model the routes of infection between co-housed birds. The presented model displays the strong correlation between housing density and *Campylobacter* prevalence, and shows how stochastic variation is the driving factor determining which strains of *Campylobacter* will emerge first within a flock. The model also shows how the system will rapidly select for phenotypic advantages, to quickly eliminate demographically-weaker strains. A global sensitivity analysis is performed, highlighting that the growth and death rate of other native bacterial species likely contributes the greatest to preventing flock outbreaks, presenting a promising approach to hypothesizing new methods of combatting disease transmission.

## 1. Introduction

*Campylobacter* is recognized as the leading cause of human gastroenteritis in the developed world (Ghareeb et al., 2013). While several transmission routes have been noted over the years (Nauta et al., 2005), poultry meat has been overwhelmingly attributed as the leading route of ingestion for humans [EFSA Panel on Biological Hazards (BIOHAZ), 2011]. An ongoing study by Public Health England has highlighted the extent to which *Campylobacter* spp. have dominated our commercial poultry. Seventy-three percent of supermarket chicken carcasses were found to contain *Campylobacter* and 7% of the outer packaging was similarly contaminated (Jorgensen et al., 2015). An estimated 450,000 people across the United Kingdom are infected every year, with 10% of these infections resulting in hospitalization (Strachan and Forbes, 2010). The immediate impact of infection is rarely fatal in the developed world, characterized by stomach cramps and diarrhea, however the resulting sequelae, while rare, are far more serious. *Campylobacteriosis* leaves the host ~100 times more likely to develop the auto-immune disorder Guillain-Barré syndrome (McCarthy and Giesecke, 2001).

While the bacteria provoke an aggressive response in human hosts, the most common species, *Campylobacter jejuni*, is considered commensal within its most common host, broiler chickens. The term “broiler” refers to any chicken bred and raised specifically for meat production. Once *Campylobacter* is present in a flock, full colonization of all birds occurs very rapidly (Evans and Sayers, 2000). From the introduction of one infected bird, it can take only a single week for an entire flock to become infected (Stern N.J. et al., 2001). The bacteria are spread via the fecal-oral route. After becoming infected, the newly-infected host broiler spends a brief period in a non-infectious incubation period, before excreting the bacteria in its fecal and cecal matter. Surrounding susceptible broilers are then exposed to this by ingesting the surrounding feed and water (Shanker et al., 1990). While the direct cause of introduction to the flock is uncertain, an exhaustive review by Adkin et al. (2006) considered that horizontal transmission is by far the most likely route, primarily being brought into a susceptible flock from some other source on the farm, such as the enclosures of other farm animals. This is as opposed to vertical transmission from breeder flocks, which are themselves often fully colonized by *Campylobacter* spp. Nevertheless, there may be a combination of both routes of entry into a flock, which deserves greater consideration.

*Campylobacter* is very rarely observed to colonize the gut of very young chickens (0–2 weeks of age) (Newell and Wagenaar, 2000). This is theorized to be the result of a supply of innate maternal antibodies acquired during a pre-laying period. This immunity has been shown to have significant bactericidal properties (Sahin et al., 2001).

Despite numerous intervention measures being trialed and employed on farms, little impact has been seen in reducing outbreak incidence (Hermans et al., 2011). This is due in part to the aggressive rate of proliferation once *Campylobacter* has entered a flock, coupled with persisting uncertainty in the exact route of primary infection. Specifically designed prevention methods are also marred by genetic variation and plasticity of *Campylobacter* spp. (Tresse et al., 2017).

Of increasing concern is the growing trend of antimicrobial resistance in *campylobacteriosis* outbreaks. Roughly 90% of the antibiotics applied in agriculture are used only to promote growth or as prophylactic agents, as opposed to being used to treat infection (Khachatourians, 1998). This overzealous use has been a major contributing factor to the continuing spread of antibiotic resistance. Ge et al. (2003) conducted a study showing that 94% of tested raw chicken samples were resistant to at least one of seven antibiotics being tested, 54% of which showed resistance to erythromycin, the antibiotic most commonly used to treat *campylobacteriosis*. These antimicrobial-resistant strains cause more prolonged and severe illness in humans (Travers and Michael, 2002) and create a scenario where *in-vitro* susceptibility testing may be necessary before any drugs may be prescribed.

Despite a wealth of empirical investigations, there is a lack of knowledge synthesizing these empirical findings through theoretical modeling frameworks. Only two studies have considered a theoretical approach to understanding *Campylobacter* spp. outbreaks; Hartnett et al. (2001) and Van Gerwe et al. (2005), who built a basic susceptible-infected (SI) model and a probabilistic model, respectively. Both frameworks only consider a model on the scale of a flock through basic susceptible-infected interactions. These approaches are not sophisticated enough to develop any meaningful theories on *Campylobacter* dynamics, as they do not represent or convey any specific interbacterial actions by *Campylobacter* populations. The lack of modeling approaches is likely due in part to the inherent challenges of mathematically simulating a gut microbiome. Over 100 different bacterial genera have been isolated from the intestines of chickens (Pan and Yu, 2014), all with a range of individual ecological interactions with one-another. Questions must then be asked regarding how to simulate the temporal and spatial impact of gut motility on the development of a microbial community. Despite these challenges, simplified models of stochastic differential equations have proved effective in capturing the often frenetic bacterial population dynamics within the gut (Wiles et al., 2016).

Here, we introduce a framework of stochastic differential equations that captures the basic interactions that are known to be observed within the broiler gut. Using this framework we simulate the propagation of multiple strains of *Campylobacter* through multiple birds in a flock. In the analysis presented below we observe key dynamical behavior commonly observed through experimentation, which can now be mechanistically explained using this theoretical framework. The theoretical insights derived from this model can be used to refine current hypotheses regarding *Campylobacter* transmission and inform future experimental and control efforts. The model will likely be of use to experimentalists and risk assessors in theorizing the impact of potential new disease prevention methods on the bacterial transmission dynamics.

## 2. Modeling Frameworks

### 2.1. Deterministic Model

Before presenting the stochastic differential equation framework, we begin by introducing the underlying deterministic core of the framework and the particular interactions modeled. Consider four variables to describe the bacterial populations within a broiler's digestive tract. *C*, the proportion of a single bird's gut flora made up of *Campylobacter*. *B*, the proportion of the gut flora made up of other bacterial species competing for space and resources. *P*, the proportion of the gut containing host defense peptides (HDPs) (this may also be interpreted as other plausible forms of host autoimmune response). Lastly, *M*, the proportion of the gut containing innate maternal antibodies. These all take values ranging such that 0 ≤ *C, B, P, M* ≤ 1. The set of ODEs describing the dynamics follows:

(1) $$\begin{array}{l} \frac{\text{d}C}{\text{d}t}=r_{1}C\left(1-\left(C+\alpha_{1}B\right)\right)-\gamma CP-d_{1}C-\beta CB-\sigma CM, \end{array}$$

(2) $$\begin{array}{l} \frac{\text{d}B}{\text{d}t}=r_{2}B\left(1-\left(B+\alpha_{2}C\right)\right)-d_{2}B, \end{array}$$

(3) $$\begin{array}{l} \frac{\text{d}P}{\text{d}t}=\xi CP\left(1-P\right)-d_{3}P, \end{array}$$

(4) $$\begin{array}{l} \frac{\text{d}M}{\text{d}t}=-d_{4}M. \end{array}$$

All rate constants are defined below in Table 1. Model boundedness is shown in Appendix S1 (Supplementary Material). The first term (*r*_1_*C*(1 − (*C* + α_1_*B*))) in Equation (1) describes the logistic growth of *Campylobacter* to a relative carrying capacity of 1, while in competition with other bacteria *B*. Competition for resources is the key to success within the gut. *Campylobacter* is known to be an effective colonizer (Stahl et al., 2012), as it is very effective at drawing zinc and iron from its environment (Gielda and DiRita, 2012; Raines et al., 2016). The second term (γ*CP*) in Equation (1) models the inhibitory effect of host defense peptides, *P*. These peptides are created in response to challenge by *Campylobacter*, as shown by Cawthraw et al. (1994). The third term (*d*_1_*C*) of Equation (1) simply describes the natural death rate of *Campylobacter*. The fourth term (β*CB*) simulates an important interbacterial interaction; that some of the most abundant competing bacteria in the microbiome have an inhibitory effect on *Campylobacter* (Schoeni and Doyle, 1992). The final term (σ*CM*) of Equation (1) represents the strong bactericidal abilities of the bird's maternal antibodies. All chickens hatch with an initial supply of antibodies that depletes over time, gone by about 3 weeks of age (Sahin et al., 2001) (most broilers are slaughtered at 5 or 6 weeks of age, however some organic and free-range flocks are slaughtered at approximately 8 weeks). These antibodies have a strong inhibitory effect on *Campylobacter*, and many studies are unable to detect *Campylobacter* (by culture methods) in birds under 2 weeks of age under commercial conditions (Sahin et al., 2015). However, forced inoculation of high-quantities of *Campylobacter* soon after hatching can still result in expression of the bacteria (Welkos, 1984).

**Table 1:** Model parameters and baseline values.

**Expression**	**Description**	**Value**
*r*_*C*_*j*__	Growth rate for *Campylobacter* strain *j*.	0.3009
*r*_2_	Growth rate for other bacteria (*B*).	0.1407
α_1_	*Campylobacter* competition coefficient.	0.9744
α_2_	Other bacteria competition coefficient.	1
γ_*C*_*j*__	Rate of inhibition by host defense peptides (*P*) on *Campylobacter* strain *j*.	0.6358
ξ_*j*_	Rate of host defense peptide growth in response to *Campylobacter* strain *j*.	0.7411
*b*	Rate of broiler shedding *Campylobacter* into the environment, *E*_*j*_.	10
Ω	Total environmental carrying capacity of *Campylobacter*.	200, 000^*^
*d*_*C*_*j*__	Death rate of *Campylobacter* strain *j*.	0.0185
*d*_2_	Death rate of other bacteria.	0.0212
*d*_3_	Decay rate of host defense peptides.	0.0463
*d*_4_	Decay rate of maternal antibodies.	0.0046
*d*_5_	Death rate of *Campylobacter* in the environment.	0.05
β_*C*_*j*__	Rate of inhibition by other bacteria on *Campylobacter* strain *j*.	0.0276
σ_*C*_*j*__	Rate of inhibition by maternal antibodies on *Campylobacter* strain *j*.	0.0661
η_*C*_*j*__	Scaling factor applied to stochastic *Campylobacter* growth in the gut.	0.01
η_*B*_*C*__*j*__	Scaling factor applied to stochastic *Campylobacter* inhibition by other competing bacteria.	0.0847
η_2_	Scaling factor applied to stochastic competing bacteria (*B*) growth.	0.01
η_3_	Scaling factor applied to stochastic host defense peptide (*P*) growth.	0.01
η_4_	Scaling factor applied to stochastic maternal antibody (*M*) decay.	0.01
η_5_	Scaling factor applied to stochastic *Campylobacter* growth in the environment.	0.01

*Descriptions for all parameter values appearing in the final stochastic model, Equations (14–18). Baseline values are given, used for model validation and simulation case studies. Values were calculated by using simulated annealing to identify a parameter set that best fits the experimental data of Achen et al. (1998) (Figure 2). See Appendix S2 in Supplementary Material for an explanation of simulated annealing*.

* *Ω value is dependent on the experiment specifics for model validation, but flock case studies consider a flock of 400 chickens, and an Ω value of 200,000*.

Equations (2), (3), and (4) follow a similar logic to Equation (1). Other bacteria, *B*, grow in competition with *Campylobacter* to a carrying capacity. Defense peptides, *P*, grow in response to *Campylobacter* expression (not in competition for resources), and the population of maternal antibodies, *M*, does not grow. All variables decay at a rate proportional to their respective populations.

Note that the above model could be reduced by amalgamating terms in Equations (1) and (2), however we choose to keep these separate to (i) keep biological processes clearly defined, and (ii) make further model development and sensitivity analyses clearer.

**Figure 1:**
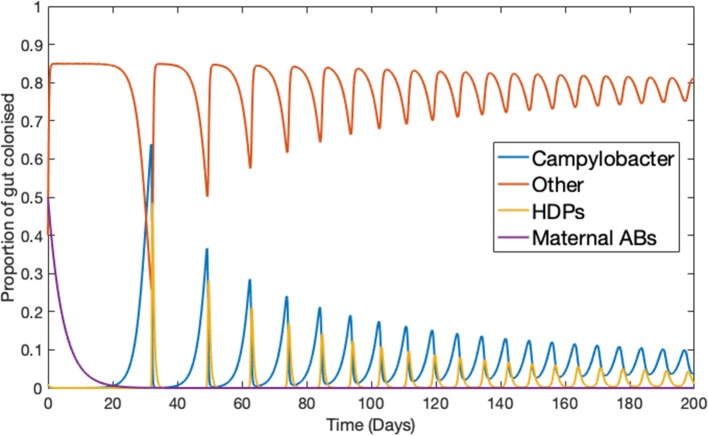
Deterministic model for one chicken. An example of the typical dynamical behavior observed for simulations of Equations (1–4). Parameters defined in Table 1.

Ignoring the trivial cases of complete domination by either *C* or *B*, the basic dynamical behavior observed for this simplified model is illustrated in Figure 1. Notably, *Campylobacter* is absent from the microbiome until the maternal antibody population has been exhausted. At this point a sudden, temporary, surge in the population of *Campylobacter* is observed. This phenomena is due to the very low population of HDPs, caused by the strong effect of the initial maternal antibodies. The HDP population then quickly rises to meet this sudden challenge, bringing the *Campylobacter* population back to a lower level in an oscillating manner, where it eventually reaches a steady-state equilibrium. This behavior is commonly observed in experimental studies (Achen et al., 1998; Newell and Fearnley, 2003).

From this simple core of four equations we adapt the model to allow for *N* unique strains of *Campylobacter*, by describing each strain as a separate variable. Equation (1) is repeated for each individual strain, while altering the growth rate terms to reflect the fact that all strains will also be in competition with one another. This alteration is represented by the following set of ODEs:

(5) $$\begin{array}{l} \frac{\text{d}C_{j}}{\text{d}t}=r_{C_{j}}C_{j}\left(1-\left(\overset{N}{\underset{j=1}{\sum }}C_{j}+\alpha_{1}B\right)\right)-\gamma_{C_{j}}C_{j}P-d_{C_{j}}C_{j}\\ \;-\beta_{C_{j}}C_{j}B-\sigma_{C_{j}}C_{j}M, \end{array}$$

(6) $$\begin{array}{l} \frac{\text{d}B}{\text{d}t}=r_{2}B\left(1-\left(B+\alpha_{2}\overset{N}{\underset{j=1}{\sum }}C_{j}\right)\right)-d_{2}B, \end{array}$$

(7) $$\begin{array}{l} \frac{\text{d}P}{\text{d}t}=\overset{N}{\underset{j=1}{\sum }}\xi_{j}C_{j}P\left(1-P\right)-d_{3}P, \end{array}$$

(8) $$\begin{array}{l} \frac{\text{d}M}{\text{d}t}=-d_{4}M. \end{array}$$

Here *C*_*j*_ represents the *j*th strain of *Campylobacter*, where *j* ∈ {1, 2, …, *N*}, and *N* is the total number of strains. As such this adjusted model is composed of *N* + 3 variables. The next alteration is to allow for multiple birds and the ability for *Campylobacter* to move from one bird to another. This is done by repeating the *N* + 3 equations presented in Equations (5)–(8) for each bird, and introducing new variables to display the saturation of *Campylobacter* strains in the shared living space.

As such, the newly adjusted model to describe the population dynamics of *N* strains of *Campylobacter* within *L* broilers, is written as,

(9) $$\begin{array}{l} \frac{\text{d}C_{ij}}{\text{d}t}=r_{C_{j}}C_{ij}\left(1-\left(\overset{N}{\underset{j=1}{\sum }}C_{ij}+\alpha_{1}B_{i}\right)\right)-\gamma_{C_{j}}C_{ij}P_{i}-d_{C_{j}}C_{ij}\\ \;-\beta_{C_{j}}C_{ij}B_{i}-\sigma_{C_{j}}C_{ij}M_{i}+a\frac{E_{j}}{\Omega }, \end{array}$$

(10) $$\begin{array}{l} \frac{\text{d}B_{i}}{\text{d}t}=r_{2}B_{i}\left(1-\left(B_{i}+\alpha_{2}\overset{N}{\underset{j=1}{\sum }}C_{ij}\right)\right)-d_{2}B_{i}, \end{array}$$

(11) $$\begin{array}{l} \frac{\text{d}P_{i}}{\text{d}t}=\overset{N}{\underset{j=1}{\sum }}\xi_{j}C_{ij}P_{i}\left(1-P_{i}\right)-d_{3}P_{i}, \end{array}$$

(12) $$\begin{array}{l} \frac{\text{d}M_{i}}{\text{d}t}=-d_{4}M_{i}, \end{array}$$

(13) $$\begin{array}{l} \frac{\text{d}E_{j}}{\text{d}t}=\overset{L}{\underset{i=1}{\sum }}bC_{ij}\left(1-\frac{E_{j}}{\Omega }\right)-d_{5}E_{j}. \end{array}$$

Here then, *C*_*ij*_ represents the proportion of the *i*th broiler's gut bacteria which is composed of *Campylobacter* strain *j*. *B*_*i*_ is the proportion of the *i*th broiler's gut bacteria made up of other bacterial species competing for space and resources. *P*_*i*_, the proportion of the *i*th broiler's gut containing host defense peptides. *M*_*i*_ is the proportion of the *i*th broiler's gut containing innate maternal antibodies. Here *i* ∈ {1, 2, …, *L*}, where *L* is the total number of broilers. *E*_*j*_ represents the amount of *Campylobacter* strain *j* that is currently in the flock's enclosed living space. We assume a living space of fixed size shared by all broilers. As such, Ω represents this total size, or carrying capacity for strains. The first term in Equation (13) shows that the amount of strain *j* in the environment is increased by being shed from birds that are already infected with strain *j* at a rate *b*. Note from the final term $\left(a\frac{E_{j}}{\Omega }\right)$ in Equation (9) that birds may then ingest strain *j* from the environment at a rate $\frac{a}{\Omega }$. This route of infection simulates the fecal-oral route of infection, but may be interpreted as some other intermittent transmission stage between birds. As such we do not remove *Campylobacter* from the environment (*E*_*j*_) upon an ingestion event, as the possibility of further environmental contamination is not yet understood and may indeed depend on the specific route of infection. The model is now composed of *L*(*N* + 3) + *N* equations, for *N* strains of *Campylobacter*, and *L* individual broilers.

### 2.2. Stochastic Model

While several important biological phenomena can be discovered and better understood with the model in its current, deterministic, form, there are key reasons to pursue a stochastic framework. First, having one variable alone to represent the multitudes of bacterial species that make up the constantly-evolving gut microbiome is, of course, a significant simplification. In practice, these other bacterial species competing with *Campylobacter* will be constantly changing, both in resurgences of population and in how they interact with *Campylobacter*. Adding stochastic elements to these populations and interactions is a small step toward capturing some of this more unpredictable behavior. Indeed the biomass of *Campylobacter* measurable in fecal and cecal matter has been observed to fluctuate widely (Morishita et al., 1997; Achen et al., 1998). Secondly, the density dependent assumptions made when formulating the initial deterministic model, that is that interaction rates are directly proportional to the variable populations, are assumptions that break down for smaller populations. The simulations undertaken often display bacterial populations at very small quantities, especially in the initial period dominated by maternal antibodies. A stochastic system behaves very differently under these circumstances and means that the model is more likely to display cases of strain extinction, a phenomena that the deterministic model cannot capture. Indeed, the very nature of *Campylobacter* infections is one that is often described in the language of probability. The all-or-nothing nature of flock infections means that we often must ask what measures can reduce the likelihoods of infections, rather than the magnitude. Through a stochastic framework we explore multiple realizations of potential outcomes, and investigate reducing the likelihood of outbreaks in a flock of broilers. The case studies presented below highlight the need for a stochastic modeling approach to accurately capture the multi-strain dynamics of *Campylobacter* within the gut. This approach allows for the simulation of turn-over and resurgence of dominant strains, an experimentally observed behavior (Colles and Maiden, 2014) that cannot be captured by a deterministic system.

For the stochastic framework, Equations (9–13) are adjusted to the following set of stochastic differential equations,

(14) $$\begin{array}{l} \text{d}C_{ij}=[r_{C_{j}}C_{ij}\left(1-\left(\sum_{j=1}^{N}C_{ij}+\alpha_{1}B_{i}\right)\right)-{\text{γ}}_{C_{j}}C_{ij}P_{i}-d_{C_{j}}C_{ij}\\ \;-\beta_{C_{j}}C_{ij}B_{i}-\sigma_{C_{j}}C_{ij}M_{i}+a(E_{j})]\text{d}t\\ \;+\left[\eta_{C_{j}}C_{ij}+{\text{λ}}_{j}(t)-\eta_{BC_{j}}C_{ij}B_{i}\right]\text{d}W_{t}, \end{array}$$

(15) $$\begin{array}{l} \begin{array}{l} \text{d}B_{i}=\left[r_{2}B_{i}\left(1-\left(B_{i}+\alpha_{2}\sum_{j=1}^{N}C_{ij}\right)\right)-d_{2}B_{i}\right]\text{ d}t\\ \;+\;[\eta_{2}B_{i}]\;dW_{t}, \end{array} \end{array}$$

(16) $$\begin{array}{l} dP_{i}=\left[\overset{N}{\underset{j=1}{\sum }}\xi_{j}C_{ij}P_{i}\left(1-P_{i}\right)-d_{3}P_{i}\right]\text{ d}t+\left[\eta_{3}P_{i}\right]\text{d}W_{t}, \end{array}$$

(17) $$\begin{array}{l} dM_{i}=\left[-d_{4}M_{i}\right]\text{ d}t+\left[\eta_{4}M_{i}\right]\text{ d}W_{t}, \end{array}$$

(18) $$\begin{array}{l} dE_{j}=\left[\overset{L}{\underset{i=1}{\sum }}bC_{ij}\left(1-\frac{E_{j}}{\Omega }\right)-d_{5}E_{j}\right]\text{ d}t+\left[\eta_{5}E_{j}\right]\text{d}W_{t}, \end{array}$$

where λ_*j*_(*t*) is defined by;

$${\text{λ}}_{j}(t)=\{\begin{array}{ll} 0, & \text{if }C_{ij}(t)\text{=0}.\\ 2.873\times 10^{-4}, & \text{otherwise}. \end{array}$$

and where *a*(*E*_*j*_) is defined by;

$$a(E_{j})=\{\begin{array}{ll} 0.015, & \text{if }X<\frac{E_{j}}{\Omega }\text{for random variable }X\sim U(\text{0},\text{1}).\\ 0, & \text{otherwise}. \end{array}$$

Here *W*_*t*_ denotes a Wiener process (standard Brownian motion process). These stochastic increments are scaled by the respective population size and constants η_2_ through to η_5_. These constants dictate the variance of their respective Wiener processes, defining the range of stochasticity attributed to the growth rate of their respective variables. The changes and additions shown in Equation (14) warrant further explanation. The sixth term (*a*(*E*_*j*_)) in Equation (14) (the last of the deterministic terms), has been changed from a constant rate of ingestion from the environment, as seen in Equation (9), to instead have ingestion modeled by a chance to ingest *Campylobacter* depending on the amount of that strain in the environment, *E*_*j*_. The greater *E*_*j*_ is, the more likely it is for ingestion to occur.

The eighth term [λ_*j*_(*t*)] in Equation (14) is a stochastic term independent of the population of *C*_*ij*_. This is introduced to allow for the possibility of extinction events, should the population of *C*_*ij*_ reach a particularly low threshold. This threshold is decided by the value taken by λ_*j*_(*t*), in this case 2.873 × 10^−4^. As with all other parameter values displayed in Table 1, the values used in the expressions λ_*j*_(*t*) and *a*(*E*_*j*_) are calculated through model validation against the studies presented below in section 2.3. Finally, the ninth term of Equation (14) considers stochasticity surrounding the interactions between *C*_*ij*_ and the competing bacteria *B*_*i*_. This term allows for instances when the particular biodiversity and spatial structure of the gut microbiome may be more inhibitory toward *Campylobacter*, or perhaps actually assisting its growth instead.

Several interesting dynamical behaviors can be observed using this model, which are highlighted through some specific question-led case studies. Table 1 defines all parameters presented in the final stochastic model [(14–18)] as well as a baseline of parameter values that were used in model validation against real world data sets (presented below). The model is constructed to an arbitrary timescale, however the parameter values given in Table 1 ensure that multiple oscillations in the *Campylobacter* population can be observed in the below case studies, a phenomena observed in the lifespan of broilers (Morishita et al., 1997).

Note that throughout we have chosen to use a *Campylobacter* competition coefficient of α_1_ = 0.9744 < 1. This choice is justified in that bacterial populations can inhabit multiple intestinal niches that cannot be colonized by other competing bacteria. Indeed competitive exclusion therapies have been far less effective in tackling *Campylobacter* compared to other foodborne illnesses such as *Salmonella* (Stern N. et al., 2001). The deterministic model is solved using the ode45 solver, a fifth-order Runge-Kutta method in Matlab. The stochastic model is solved numerically using the Euler-Maruyama method (Higham, 2001) with *N* = 2^14^ timesteps, also programmed in Matlab. The code used to produce all figures presented is available at: https://osf.io/b3duc/.

We also note that while the model is general enough that it is not specific to any one species of *Campylobacter*, two of the three datasets that were used to tune our model parameter values were from studies unique to the most common species, *Campylobacter jejuni* (Achen et al., 1998; Stern N.J. et al., 2001). As such we may expect slightly different parameter values to be applicable for other species of *Campylobacter*. However, from testing the model, the results we present below are ones that are generally observed for a range of parameter values, and as such are relevant findings to the whole genus.

### 2.3. Model Validation

**Figure 2:**
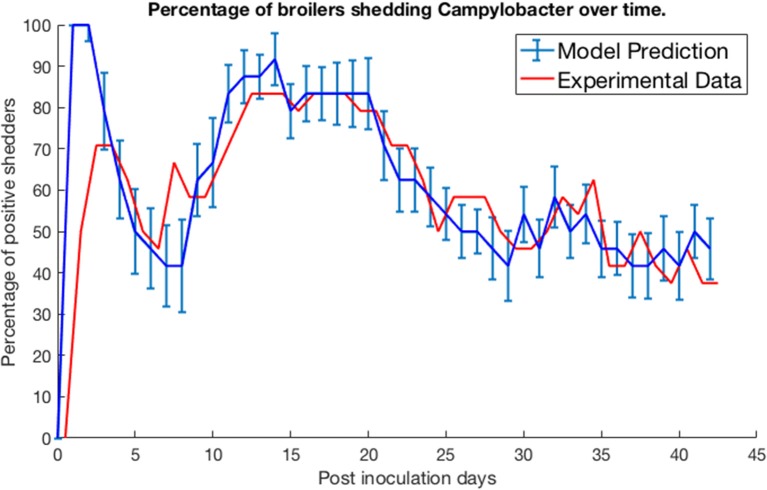
Model validation against data of Achen et al. (1998). The percentage of a group of isolated broilers shedding *Campylobacter* across several weeks following inoculation.

We test our model by comparing its predictions against three experimental studies on *Campylobacter* expression and spread. Firstly, we consider the work of Achen et al. (1998). Achen et al. performed an experiment with 24 broilers, who were kept in individual, isolated wire-bottomed cages. Birds were confirmed as free of *Campylobacter* before being inoculated with a *C. jejuni* suspension. A cloacal swab was then obtained from each bird every day for 42 days, to monitor whether or not each bird was shedding *Campylobacter*. Figure 2 shows their experimental results alongside the predictions made by our model.

Specifically, the blue line represents the modal value of the percentage of the 24 birds shedding across a thousand simulations, with error bars depicting the standard deviation across these simulations. Achen et al. (1998) also reports how most birds would shift from phases of positive shedding to negative shedding, a phenomena also captured by the oscillating behavior displayed by the model. Sampling via culture methods like those performed in this experiment is prone to false-negative results for samples with very low quantities of *Campylobacter* (Acke et al., 2009). Therefore, for this model validation, we considered a broiler as being clear of *Campylobacter* if its proportion of *Campylobacter* (variable *C*) was below 0.005. This was considered a more accurate measure to correspond with the experimental data. While our model was constructed to an arbitrary timescale, comparing to this real-world data set it was found that our time axis is best rescaled by a factor of 0.021 to align with the measure of days used in these studies. This corrected timescale is used for all subsequent case studies within this paper.

**Figure 3:**
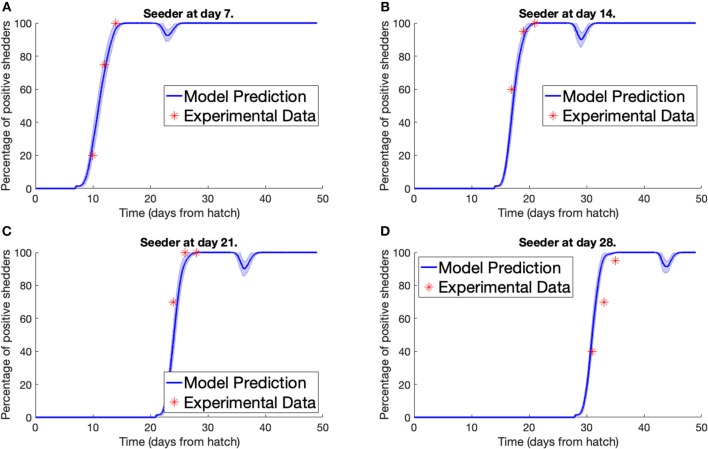
Model validation against data of Stern N.J. et al. (2001). The percentage of a flock of broilers shedding *Campylobacter* across several weeks after introduction of a *Campylobacter*-positive seeder bird at **(A)** 7 days, **(B)** 14 days, **(C)** 21 days, **(D)** 28 days.

Secondly, we consider the experiment conducted by Stern N.J. et al. (2001). Multiple separate pens were prepared, each containing 70 broilers, all free of *Campylobacter*. A *Campylobacter*-positive seeder bird was then added to the flock. Different pens had seeder birds introduced at different points in time. Three, five, and seven days after a seeder bird was introduced, a sample of chickens were tested for *Campylobacter* to estimate the percentage of the flock that was currently *Campylobacter*-positive. We plot our model predictions against Stern et al.'s experimental data below in Figure 3. To match the housing density of the experiment, a value of Ω = 45, 369 was used for the model. An error band is plotted around our model prediction displaying the standard deviation of values across 100 simulations.

**Figure 4:**
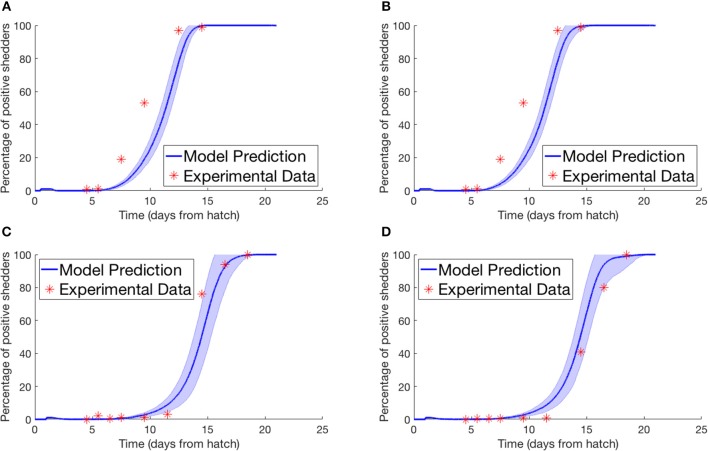
Model validation against data of Van Gerwe et al. (2005). The percentage of a flock of broilers shedding *Campylobacter* across several weeks after introduction of a *Campylobacter*-positive seeder bird. **(A,B)** Seeder bird introduced at day of hatch, samples collected via cloacal swab, **(C,D)** seeder bird introduced one day after hatch, samples collected via fresh fecal droppings.

Lastly we simulated the experiment performed by Van Gerwe et al. (2005). Four flocks of 400 birds were set up in individual enclosures from day of hatch. Four birds in each flock were then inoculated with a *Campylobacter* suspension and returned to the flock. Birds were then sampled from each flock throughout the next few weeks to record the percentage of flock infection. Figure 4 plots their experimental data against our model prediction. For the experiments shown in Figures 4A,B, the four seeder birds were inoculated at day of hatch, and chickens were sampled by cloacal swabbing. For the experiments shown in Figures 4C,D, the seeder birds were inoculated one day after hatch, and the flock was analyzed by collecting fresh fecal samples.

## 3. Simulations

We now use a series of (simulated) case studies to investigate key dynamical behaviors and predictions from the model.

### 3.1. Staggered Strain Infection

**Figure 5:**
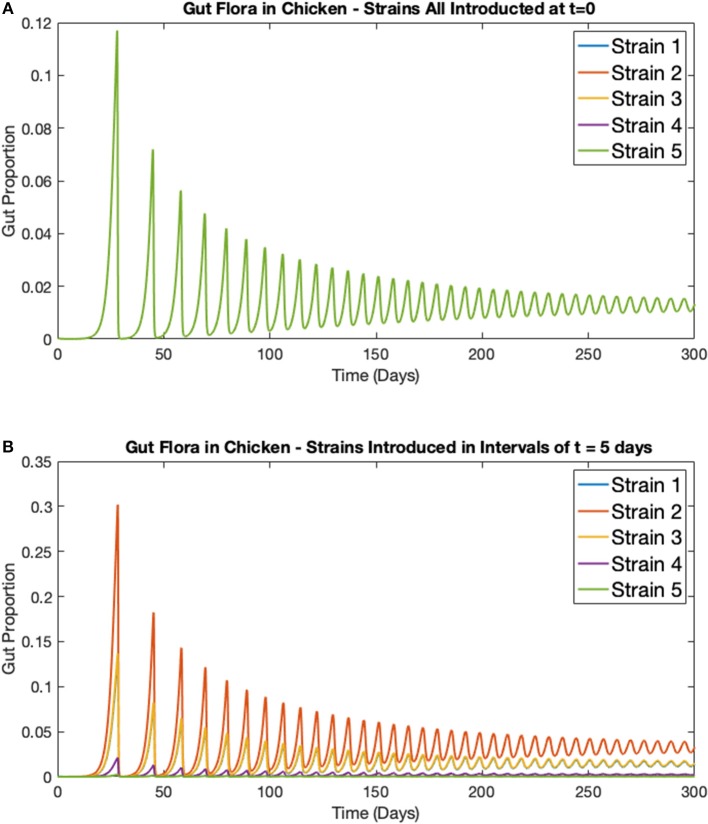
Simulations of multiple *Campylobacter* strains within one broiler. Population growth of five strains of *Campylobacter* within one broiler that are **(A)** all introduced at *t* = 0 days **(B)** introduced in intervals of *t* = 5 days. Strains are initialized at *C*_*j*_(*t*) = 0.0001 at their respective time of introduction. Other variables are initialized at *B*(0) = 0.4, *P*(0) = 0.01, and *M*(0) = 0.5. Note that the single green line in Figure 5A is due to overlap, all five strains exhibit the exact same dynamical behavior, as would be expected.

In this first example, the deterministic model for multiple strains in one broiler (Equations 5–8) is considered. Five strains of *Campylobacter* within one chicken are simulated, all with the exact same respective rate constants as shown in Table 1. Figure 5A shows the results when all five strains are introduced at *t* = 0 days, with the same initial inoculation amount of *C*_*i*_(0) = 0.0001. Figure 5B shows instead when each strain is introduced in intervals of *t* = 5 days. Therefore, only strain 1 is introduced at *t* = 0 days, strain 2 is introduced at *t* = 5 days and so on until finally strain 5 is introduced at *t* = 20 days. In both cases the other three variables are initialized at *B*(0) = 0.4, *P*(0) = 0.01, and *M*(0) = 0.5.

While the maternal antibodies (*M*) are not plotted on these figures, they approach 0 at approximately *t* = 20 days, as can be seen by the following surge in *Campylobacter* populations following this point in Figure 5. While, unsurprisingly, all strains perform identically in Figure 5A (where strains are initialized at the same point in time), a more curious dynamic is observed in Figure 5B. The strain that performs best and exists at the highest proportion in the staggered release example is strain 2, the second strain to be introduced. The reason for this is that strain 1, present at *t* = 0 days, is initially suppressed by the maternal antibodies (parameter *M*), reducing the proportion of strain 1. As a result, when strain 2 is introduced, it is able to capitalize on the severely reduced amount of strain 1, and the reduced amount of maternal antibodies, to quickly grow and dominate the competitive space. Strain 2's increased presence then puts future strains at a disadvantage as it has already had the opportunity to establish itself within the gut. These results suggest that dominant *Campylobacter* strains can prevent new strains from taking hold. Moreover, there is an optimal point in time for inoculation to occur for a strain to become dominant, as shown in Figure 5B where strain 2 is consistently occupying a higher proportion of the gut than other strains.

### 3.2. Stochastic Model—One Strain in One Broiler

**Figure 6:**
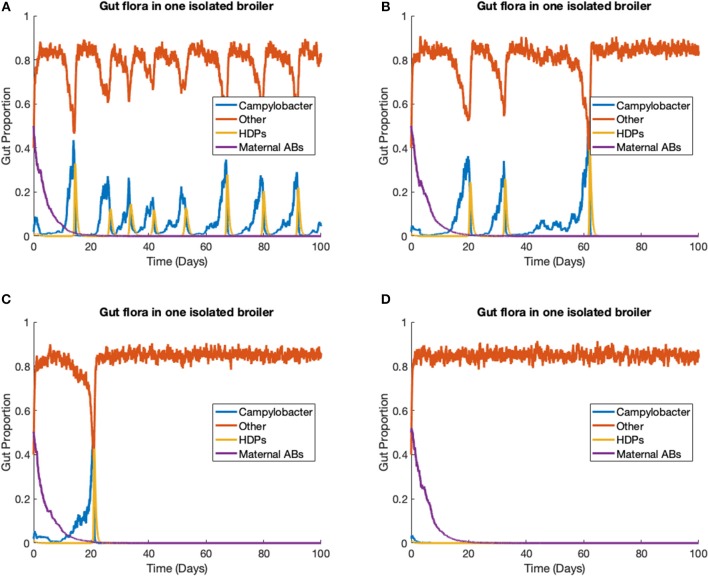
Stochastic simulations of one *Campylobacter* strain within one broiler. **(A–D)** Four different realizations of a stochastic model simulating one strain of *Campylobacter* within one isolated broiler.

The stochastic model (Equations 14–17) is run to simulate one strain of *Campylobacter* within one broiler. In this scenario, we ignore the environmental variable *E* (Equation 18), as its input is negligible for only one broiler. The rate constants are kept at the same values as used previously, defined in Table 1, with the additions of the stochastic variance scaling rate constants, parameters that limit the variance of the stochastic additions. These are set as η_*C*_*j*__ = η_2_ = η_3_ = η_4_ = 0.01, and η_*B*_*C*__*j*__ = 0.0847. η_*B*_*C*__*j*__ is set higher than the other stochastic rate constants to capture the greater unpredictability surrounding these bacterial interactions. Four different realizations of this model are presented in Figure 6, all initialized at *C*(0) = 0.02, *B*(0) = 0.4, *P*(0) = 0.01, *M*(0) = 0.5.

Empirical studies measuring the amount of *Campylobacter* in the fecal matter of isolated broilers have shown a spectrum of results. Some broilers display sustained high populations, others express initial peaks followed by great reduction and potentially later resurgence, and sometimes extinction cases are observed (Achen et al., 1998). All these dynamical behaviors can be observed in different realizations of this model (Figure 6). Figure 6A shows an instance where a broiler is consistently infected and shedding into the environment, unable to effectively clear the *Campylobacter* from its system. Figure 6B instead shows an instance where a broiler has multiple periods of high infection and shedding, before being able to clear the infection. Figure 6C shows an instance where after one initial peak in *Campylobacter* expression, a broiler is able to quickly clear infection. Finally, Figure 6D shows an instance where the broiler successfully clears *Campylobacter* at the initial point of inoculation. All these realizations are run with the same parameters given in Table 1, demonstrating the benefit of a stochastic framework being able to better capture the more diverse range of possible events. This broad array of dynamical profiles is not observed in commercial broiler flocks, a phenomena that is demonstrated in the following case study.

### 3.3. Stochastic Model—One Strain in Multiple Broilers

**Figure 7:**
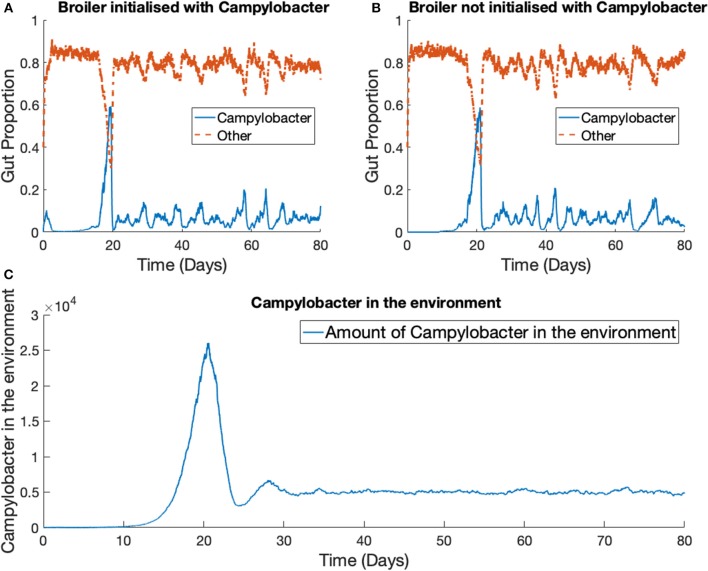
Stochastic simulations of one *Campylobacter* strain within multiple broilers. The proportion of a broiler's gut containing *Campylobacter* for **(A)** a broiler in the flock initialized with a small proportion of *Campylobacter* **(B)** a broiler in the flock initialized with no *Campylobacter*. **(C)** Shows how much of the environment (total size of 200,000) contains *Campylobacter*. This is variable *E* in the model.

The previous scenario is now extended to consider multiple broilers. Figure 7 presents the results for one *Campylobacter* strain in a flock of 400 broilers. We use the parameter values stated in Table 1. The total size of the enclosure, or the carrying capacity of *E*, is set at Ω = 200, 000. This value is considered in cm^2^, and so with 400 broilers, translates to 500 cm^2^ per broiler. EU directive 2007/43/CE states that broilers may never be stocked at more than 42 kg/m^2^ (Council of European Union, 2007). Assuming a targeted bird weight of 1.5 kg, this translates to 357 cm^2^ per bird. This simulation models slightly more space allowed to each bird than the limit. The death rate of *Campylobacter* in the environment is set at *d*_5_ = 0.05, higher than the death rate within a broiler as, despite their many survival mechanisms (Murphy et al., 2006) *Campylobacter* is susceptible to many exterior environmental stresses (Park, 2002) and is exceptionally fragile outside of its host. The simulation began with no *Campylobacter* in the surrounding environment [*E*(0) = 0] and the other initial conditions are set the same as for the previous example, with the exception that two of the 400 broilers start with an initial condition of *C*_1_(0) = *C*_2_(0) = 0.02, while the others are initialized without any *Campylobacter*. These results are shown in Figure 7.

While birds who are not initialized with *Campylobacter* become infected at a slightly later time, the dynamical behavior is very similar across all birds in the flock. Multiple realizations do not display the broader spectrum of behavior observed in the one broiler case (Figure 6). The implication is that housing a greater number of birds causes more homogeneous dynamical behavior, and indeed the wide variety of *Campylobacter* expression seen in the isolated bird experiments of Achen et al. (1998) is not so commonly observed in experiments with group-housed birds (Van Gerwe et al., 2005).

### 3.4. Stochastic Model—Five Strains in Multiple Broilers

**Figure 8:**
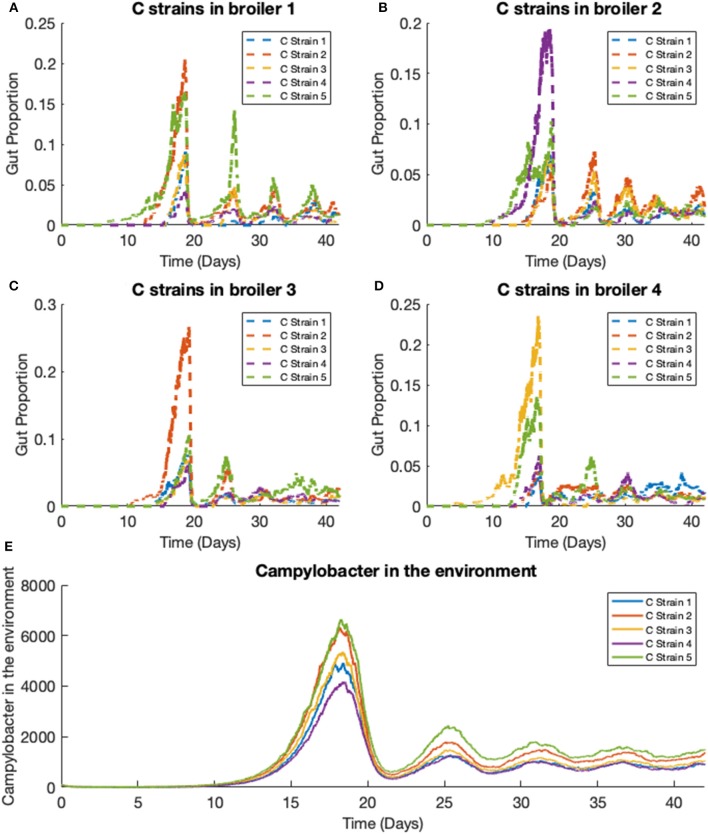
Stochastic simulations of multiple *Campylobacter* strains within multiple broilers. **(A–D)** The proportion of four different broilers' microbiomes that contain five strains of *Campylobacter*. All birds are within the same flock. **(E)** Shows how much of the environment (total size of 200,000) contains the five strains of *Campylobacter*. These are variables *E*_*j*_ in the model.

We extend the previous scenario to investigate dynamics of multiple strains. Five strains of competing *Campylobacter* are modeled within the same flock of 400 birds. The same constants are used as in the previous scenario, with each strain having identical rate constants. One key difference is that all broilers are initialized without any *Campylobacter*, instead an initial amount is present in the environment. Each strain of *Campylobacter* in the environment is initialized at *E*_1_(0) = *E*_2_(0) = *E*_3_(0) = *E*_4_(0) = *E*_5_(0) = 100. The results of this simulation are shown in Figure 8.

On average, all strains perform equally well across the flock, as shown in Figure 8E. All strains slowly converge to roughly equal amounts in the environment, reflecting an equal presence on average across all birds in the flock. However, when observing the *Campylobacter* proportions within individual broilers, one or two strains will tend to dominate early on in colonizing a broiler's gut, which can in turn prevent other strains from taking hold (seen most clearly in Figure 8D). This dynamical behavior was first observed in our deterministic simulations (see Figure 5B), however unlike in the deterministic case, stochastic events can cause dominant *Campylobacter* strains to reduce in population, presenting an opportunity for a different strain to establish itself.

**Figure 9:**
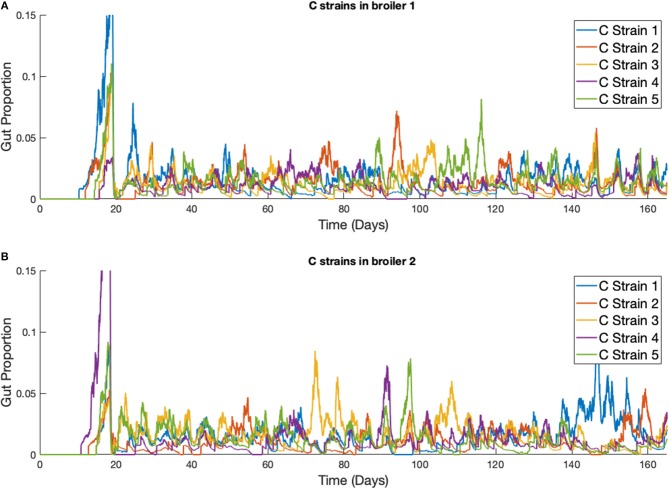
Stochastic simulations of multiple *Campylobacter* strains within multiple broilers across a greater timescale. **(A,B)** The proportion of two different broilers' microbiomes that contain five identical strains of *Campylobacter*.

This phenomena is more clearly seen if the timescale of the simulation is extended, as illustrated in Figure 9. Although the average population of strains across the flock is equal, the stochastic model shows that a single strain of *Campylobacter* tends to dominate the gut of individual broilers at any one time. Although there are brief periods where strains exist in equal amounts, eventually the balance shifts again to longer periods of dominance by one or perhaps two strains.

**Figure 10:**
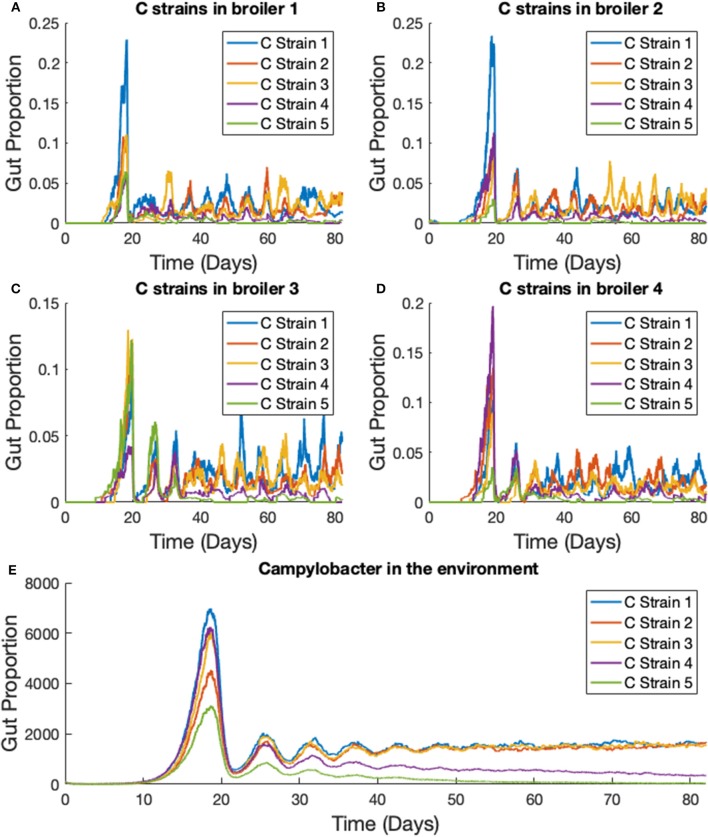
Stochastic simulations of multiple *Campylobacter* strains, differing in growth rates, within multiple broilers. **(A–D)** The proportion of four different broilers' microbiomes that contain five strains of *Campylobacter*. Strain 4 has had it's growth rate reduced from 0.3009 to 0.295 and strain 5 has had its growth rate reduced to 0.29. Strains 1, 2, and 3 have a growth rate of 0.3009. **(E)** Shows how much of the environment (total size of 200,000) contains the five strains of *Campylobacter*. These are variables *E*_*j*_ in the model.

Disadvantaged strains of *Campylobacter* are quickly eliminated. Figure 10 shows the results for a simulation where strain 4's growth rate, *r*_*C*_4__, is reduced from 0.3009 to 0.295, and strain 5's growth rate, *r*_*C*_5__, is reduced to 0.29. Strains 1, 2, and 3 are kept with a growth rate of 0.3009. As Figure 10 shows, the weaker strains are unable to outcompete the other three and are quickly eliminated. Changing other demographic parameters of a strain achieve a similar result of driving a strain to extinction, the phenomenon is not unique to only altering the growth rate. Making only very small reductions to the growth rate can result in a strain surviving at a lower average population size, although this may only be due to the time needed for extinction to occur being too long to observe in these simulations.

## 4. Sensitivity Analysis

A powerful use of this model is to conduct a robust sensitivity analysis to identify the parameters of greatest impact in driving outbreaks of *Campylobacter*. We adopt a variance-based analysis of the model, and investigate the likelihood of flocks remaining free of *Campylobacter* based on a random assignment of parameter values.

**Figure 11:**
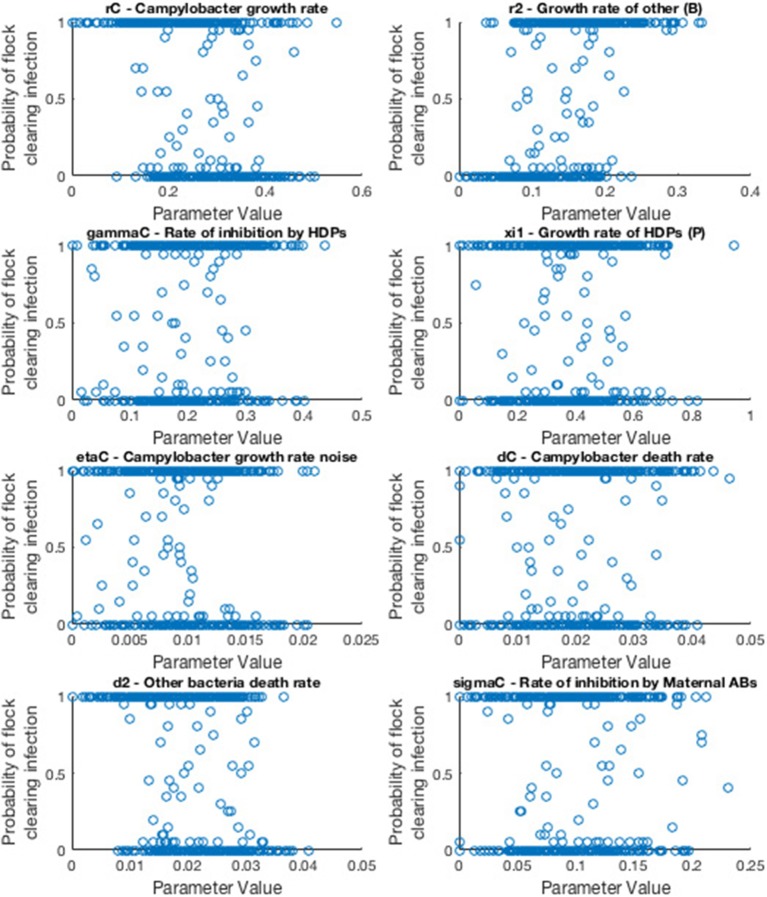
Scatter plots displaying probability of a flock clearing *Campylobacter* infection against randomly sampled parameter values. Each scatter plot depicts the results for a specific parameter value. Probability is calculated by running the model for a sampled parameter set 20 times, and recording how many of those runs resulted in the flock not becoming infected with *Campylobacter*.

We consider the case of a flock of broilers infected with a single strain of *Campylobacter*, the scenario shown in section 2.3. Model parameters are sampled randomly from a uniform range, and the model is run multiple times for these values. We then record how many of these stochastic runs resulted in the flock successfully eliminating *Campylobacter* infections, before drawing a new random sample of parameters values and repeating as necessary. Eventually we finish with a final data set which we display an example of below in Figure 11.

As such, the most “important” parameters will be the ones which exhibit a strong trend in their scatter plot. A seemingly randomly distributed scatter plot would indicate a parameter value which has little impact on our output. To report more accurately this measure we use the first-order sensitivity index, *S*_*i*_, and the total effect index, *S*_*T*_*i*__, defined as:

$$\begin{array}{l} S_{i}=\frac{V_{X_{i}}\left(E_{X_{\sim i}}\left(Y|X_{i}\right)\right)}{V\left(Y\right)},\;S_{T_{i}}=\frac{E\left(V\left(Y|X_{\sim i}\right)\right)}{V\left(Y\right)}, \end{array}$$

where *X*_*i*_ denotes parameter *i*, and *Y* denotes the model output. *X*_~*i*_ denotes the vector of all factors but *X*_*i*_. *V*(·) denotes the variance, and *E*(·) the expectation. Specifically *E*(*A*|*B*) denotes the expectation of variable A when B is held fixed. In short *S*_*i*_ will measure the changes observed in the output when parameter *X*_*i*_ is kept fixed, while *S*_*T*_*i*__ measures the changes to the output when all other parameters are kept fixed. A full derivation and explanation can be found in Saltelli et al. (2008). In short, both are values that range from zero to one, that explain the impact of a parameter on the model output. The higher the value, the more “important” the parameter is. *S*_*T*_*i*__ is considered a stronger metric, as it also considers the higher-order impact of a parameter, whereas *S*_*i*_ only considers the immediate first-order impact. As such *S*_*i*_ would be a sufficient measure for a linear model, but for a more complex model such as the one presented in this paper, *S*_*T*_*i*__ can better reveal the impact that each parameter plays. An initial sensitivity analysis was run for 20 parameters with 2, 000 parameter set samples, drawn from a quasi-random Sobol set (Saltelli et al., 2008). The results of this analysis are displayed in Table 2, and the code used to produce them is available to access at: https://osf.io/b3duc/.

**Table 2:** Sensitivity analysis of parameters in a stochastic model for one *Campylobacter* strain in a flock of broilers.

**S_*i*_**	**Parameter**	**S_*T*_*i*__**	**Parameter**
−0.0112	η_3_	−0.0031	σ_*C*_
−0.0054	η_*BC*_	−0.0026	η_4_
−0.0051	*a*	−0.0016	η_5_
−0.0017	η_*C*_	−0.0011	*d*_4_
−0.0011	η_2_	0.0056	*b*
0.0009	η_5_	0.0065	*a*
0.0020	*d*_4_	0.0114	γ_*C*_
0.0025	*b*	0.0122	ξ
0.0038	η_4_	0.0224	η_*C*_
0.0054	ξ	0.0228	η_*BC*_
0.0062	σ_*C*_	0.0378	η_3_
0.0077	γ_*C*_	0.0389	Ω
0.0085	Ω	0.0417	η_2_
0.0096	*d*_5_	0.0543	*d*_5_
0.0125	β_*C*_	0.1380	*d*_*C*_
0.0441	*d*_*C*_	0.1925	β_*C*_
0.0459	*r*_*C*_	0.2088	*r*_*C*_
0.1131	*d*_3_	0.3117	*d*_3_
0.1622	*r*_2_	0.4418	*d*_2_
0.1900	*d*_2_	0.4789	*r*_2_

*The first-order sensitivity index and total effect index is given for a sensitivity analysis of 2, 000 runs for 20 parameters. The output function considered is the probability of Campylobacter going extinct within the flock based on the given parameter set*.

Specifically, our objective function will run the stochastic model for a flock of chickens with the random parameter set drawn. If this model run results in no *Campylobacter* being present in the flock, it is considered to have successfully eliminated infection. The model is run 20 times with this parameter set, and the proportion of these 20 runs that results in an elimination of *Campylobacter* is the final output value, the “probability of flock clearing infection.”

Note that some of the values in Table 2 are negative, despite *S*_*i*_ and *S*_*T*_*i*__ being limited to being between zero and one. This is due to the computational error in estimating the value, however the ordering of parameters for these particular runs will not be affected by this error. Table 2 shows that the *S*_*T*_*i*__ values associated with most parameters ranges between 0 and 0.1. The “most important” parameters however have a wider spread of associated *S*_*T*_*i*__ values.

The main result from this analysis is that the growth, death and inhibition rates of the other bacteria present in a broiler's gut (parameters *r*_2_, *d*_2_, and β_*C*_) collectively carry the largest impact in eliminating *Campylobacter* from a flock. As such, we can begin to consider which preventative methods could best take advantage of this heightened sensitivity.

## 5. Discussion

Here, we have investigated the dynamics of *Campylobacter* across a range of model applications. Our framework reveals several key dynamics of microbial interaction that explain many experimentally observed phenomena. This presents promising new approaches to understanding and tackling this bacteria.

First, the most apparent prediction is that the *Campylobacter* population is successfully suppressed by the innate maternal antibodies (an experimentally observed phenomenon; Connerton et al., 2018), until these antibodies are eventually removed from the system. At this point an initial surge in the population of *Campylobacter* is observed, before it comes to rest at a lower level, reaching an equilibrium with the broiler's immune-response. This can be seen in all of the above figures, but most clearly in Figure 1. This initial surge creates an interesting opportunity for certain strains of *Campylobacter* to emerge as an early dominating strain. Figure 5B shows that, due to the antibacterial properties of a broiler's maternal antibodies, any strains that infect a broiler early on in its lifespan will be heavily inhibited. This creates a brief window at the point in which maternal antibodies have depleted, whereby any new strain introduced is observed to quickly colonize and dominate the gut flora, suppressing other strains (see Figure 10C). This hypothesis has been verified experimentally (Connerton et al., 2018).

The proposition of damped oscillations between *Campylobacter* population size and the host's immune-response is better reinforced by observations that host antibody populations will also oscillate in birds infected with *Campylobacter* (Cawthraw et al., 1994). This basic interaction has been experimentally observed by Achen et al. (1998), with a high degree of variability between birds. This variability is better captured by the stochastic model, as shown in Figure 6. Indeed, many birds in Achen et al.'s study are shown to clear *Campylobacter* successfully from their system, a result rarely observed on commercial broiler farms. Likewise this result was only observed in the model case of individual, isolated broilers (see Figure 6D).

Most important is the mechanism observed in Figure 7, where the broad spectrum of oscillatory behavior observed within a broiler is greatly reduced in a large flock of birds. Indeed the vast examples of individual dynamics observed in Figure 6, large oscillations and perhaps extinctions, are completely replaced by the same, homogenized dynamics seen within flock-reared birds in Figures 7A,B, as the populations of *Campylobacter* within each bird are consistently reinforced by the amount of *Campylobacter* in the environment. The wealth of experiments in monitoring flock *Campylobacter* expression for varying flock sizes means this effect can be observed taking place across multiple experiments of different flock magnitudes and densities. Morishita et al. (1997) measured the amount of *Campylobacter* in a flock of thirty birds in a sizeable pen. This flock was small enough to observe oscillating behavior in the prevalence of *Campylobacter*, and yet there do not appear to be any clear cases of birds being able to clear the bacteria from their system. Stern N.J. et al. (2001) experimented with flocks of 70 birds at a density of 15.4 birds/m^2^. A small cyclic pattern is observable in their results but there are clearly far higher incidence rates. Lastly, Van Gerwe et al. (2005) studied flocks of 400 birds housed at 20 birds/m^2^ (the same density considered in the above flock modeling), where now no cyclic patterns can be observed, and all birds quickly reach a constant state of *Campylobacter* expression. This effect is seen in Figure 7, and almost always observed in commercial farms (Stern et al., 1995; Evans and Sayers, 2000). Our work presented here is the first, to our knowledge, to be able to propose a mechanistic explanation for this observed effect, namely that the housing density of reared flocks is correlated to *Campylobacter* prevalence.

This dynamic, whereby broilers are consistently infected with *Campylobacter* due to highly contaminated living space, can also explain the observed phenomena whereby broiler breeder flocks (flocks kept for the breeding of meat birds) display a consistently lower *Campylobacter* prevalence rate than commercial broiler flocks (Colles et al., 2011). Breeder birds will regularly move between periods of testing positive and negative for *Campylobacter*, inconsistently with the state of other birds in the flock, unlike the much younger birds grown for meat which remain consistently positive. Our case studies suggest that this may be due to the lower stocking density afforded to breeder birds, as it would appear the route of infection between breeder birds is weaker than that between broilers. Our sensitivity analysis however also highlighted that the gut flora can have a strong impact on the survival of *Campylobacter*. The differences in diet and management practice for breeder birds likely results in a different variety of bacterial colonies to broilers, which could also be a cause of the differences seen between breeders and broilers in *Campylobacter* expression.

Additionally, we note that over these case studies we have seen that the outbreak dynamics are unaffected by the initial method of inoculation. There is no clear consensus yet on whether flocks are initially infected through horizontal or vertical transmission, and our model predictions show that this may not be possible to determine from flock infection dynamics. Case studies showed no difference between initialization with an infected environment, or an infected number of broilers. More specifically, Figure 7 shows clearly how the dynamic profile of a broiler that is initialized with *Campylobacter*, is not significantly different from that of a bird which is infected through the environment, representing the effect of vertical and horizontal transmission respectively.

Over time, our model shows strains of equal fitness will tend to settle at equal levels of prevalence on average across a flock (Figure 8E), a result that has also been shown experimentally (Colles and Maiden, 2014; Colles et al., 2015). However, it is very common for an individual broiler to have only one or two dominant strains against far smaller proportions of other strains (Colles et al., 2019), as our model represents in Figures 8A–D, 9. Our results show that this effect is most prominently seen early on in the chicken's lifespan, where usually only one strain will be present during the initial population surge of *Campylobacter*. Colles et al. (2019) shows that a greater diversity of strains are observable later on in a broiler's lifespan, but usually at a far lower prevalence compared to a dominating strain. Evidently, when one strain is already well-established within a chicken's gut, it is difficult for new competing strains to grow. This is due to the broiler already having a heightened level of immune response (*P*) due to the currently present strain. In the deterministic case, later strains would never be able to establish themselves as much as strains that were earlier to arrive (Figure 5B). However, in the stochastic model, there is the potential for a stochastic event to reduce the population of the currently dominating strain, and increase the population of a less-established strain.

Across the whole flock, weaker strains can be quickly outcompeted by other strains. Figure 10 shows two weaker strains (strains with lower growth rates) attempting to survive within a flock, even having a slight population peak at the optimal point of strain introduction, before eventually being forced to extinction by the other three strains. Parameter variation showed that reducing a strain's demographic parameters by a very small amount can allow it to persist still in the flock at a smaller average population than the others, but the majority of realizations would always end with weaker strains becoming extinct. Clearly this shows an environment where genetic dominance is very quickly selected for.

These results have considerable implications for biosecurity. While smaller flocks may have a very real opportunity to be protected from *Campylobacter* invasions, *Campylobacter* prevalence is far more stable in larger commercial flocks, and our model shows it to be exceptionally difficult to remove. Efforts can be made to prevent initial inoculations, but once a bacterial presence is established, it may be all but impossible to remove from a flock. Considerable improvements to biosecurity have been made in recent years, but very little impact has been observed in this having reduced *Campylobacter* incidence (Hermans et al., 2011). These measures do not reduce the speed of proliferation of the bacteria, and our results suggest that better attention to bird health is likely to have a greater effect on preventing flock infection.

This model's greatest strength is its lack of overarching assumptions. We model only the most basic bacterial interactions, all supported and verified through experimental work. Our stochastic system is capable of exhibiting a plethora of interesting dynamical interactions based on just a few known biological interactions. In moving forward with this work, the model can be used to theorize optimal methods by which to decrease the likelihood of *Campylobacter* outbreaks, and begin collaborative efforts in better explaining the evolving genetic diversity of this bacteria.

One area in which the model is admittedly lacking currently, is that it does not represent the physiological changes that occur as a bird grows. Broilers have been genetically selected over the many decades to grow excessively fast, which has been shown to have numerous concerning implications for their health (Buzała et al., 2015). This is likely to then result in differences to their auto-immune capabilities over time. More pertinently, the gut flora of a chicken is known to change and develop as the birds age (Lu et al., 2003), suggesting varying degrees of inter-bacterial uncertainty.

Our sensitivity analysis gives great insight into the optimal routes of infection prevention. Table 2 clearly shows that bolstering the growth rate and inhibition capabilities of the other bacteria populating a broiler's gut is the best way to force extinction of *Campylobacter*, primarily through suppressing *Campylobacter* at its initial appearance in a system, before it has the opportunity to propagate. As such, the sensitivity analysis suggests further exploration and experimentation into the impact of factors which would affect the gut flora of a broiler. Probiotics are a clear way of impacting the microflora (Mountzouris et al., 2007) and have shown some effect in studies into their impact on *Campylobacter* expression (Santini et al., 2010). Equally, the stressors linked with stocking density have been shown to affect the gut microflora by Guardia et al. (2011). Burkholder et al. (2008) have shown that feed withdrawal and heat stress can considerably alter and limit the gut microflora. These highlight that general bird health and welfare can be equally strong factors in determining the values of *r*_2_, *d*_2_, and β_*C*_; some of the parameters highlighted as most “important” by the sensitivity analysis. Table 2 also however highlights the importance of parameter *d*_3_, the death rate of host defense peptides. This parameter has been shown to be strongly affected by stressors such as overcrowding (Gomes et al., 2014). As such, this result would lend further support to giving greater care to the health and welfare of broilers, as the resulting improvement to host defense peptide production would have a positive impact on helping prevent *Campylobacter* outbreaks.

These caveats notwithstanding, the model presented is capable of explaining a wealth of experimentally observed *Campylobacter* population dynamics, further elucidating an urgent public health risk. We have used our framework to investigate multiple strain interactions, to understand better the spread of genotypes across a flock. Finally, we were able to use the model to highlight the factors most responsible for causing outbreaks of infection. Looking forward, this work can be used to understand better observed differences in outbreak dynamics between different farms and indeed countries, and further our goal of minimizing public exposure to this dangerous pathogen.

## Data Availability

All datasets generated for this study are included in the manuscript and/or the Supplementary Files.

## Author Contributions

TR designed the model and performed the presented case studies. TR wrote the manuscript. MB and MD supervised the project and proof-read the manuscript.

### Conflict of Interest Statement

The authors declare that the research was conducted in the absence of any commercial or financial relationships that could be construed as a potential conflict of interest.
