# Supplementary material for: A Mathematical Model of Campylobacter Dynamics Within a Broiler Flock
Source: Front Microbiol. 2019 Aug 21;10:1940. doi: 10.3389/fmicb.2019.01940 (PMC6712969; doi:10.3389/fmicb.2019.01940)
Supplement: Supplementary file 1 [file Data_Sheet_1.pdf]

Supplementary Material

S1 APPENDIX - MODEL BOUNDEDNESS

The basic deterministic core of the model is:

$$\frac{\mathrm{d}C}{\mathrm{d}t} = r_1 C\left(1 - (C + \alpha_1 B)\right) - \gamma CP - d_1 C - \beta CB - \sigma CM, \quad \text{(S1)}$$

$$\frac{\mathrm{d}B}{\mathrm{d}t} = r_2 B\left(1 - (B + \alpha_2 C)\right) - d_2 B, \quad \text{(S2)}$$

$$\frac{\mathrm{d}P}{\mathrm{d}t} = \xi CP(1 - P) - d_3 P, \quad \text{(S3)}$$

$$\frac{\mathrm{d}M}{\mathrm{d}t} = -d_4 M. \quad \text{(S4)}$$

We can show that C and B are bounded between 0 and 1. If $C = 0$, then $\frac{\mathrm{d}C}{\mathrm{d}t} = 0$. Likewise if $B = 0$, then $\frac{\mathrm{d}B}{\mathrm{d}t} = 0$. Hence neither variable is capable of decreasing below 0, and they are bounded below at 0.

If $C = 1$, then equation (19) becomes:

$$\frac{\mathrm{d}C}{\mathrm{d}t}|_{C=1} = r_1(-\alpha_1 B) - (\gamma P + d_1 + \beta B + \sigma M).$$

Since all parameters are positive, and we have shown that $B > 0$, we can conclude that $\frac{\mathrm{d}C}{\mathrm{d}t}|_{C=1} < 0$. Therefore C cannot exceed one, and is bounded above. We can likewise show the same for B.

Likewise equation (21) shows that when $P = 0$, $\frac{\mathrm{d}P}{\mathrm{d}t} = 0$. When $P = 1$, $\frac{\mathrm{d}P}{\mathrm{d}t} = -d_3$. Hence P is also bounded between 0 and 1.

M is clearly be bounded as it is only decreasing down to 0, and we initialise it at a value less than 1.

There is however some issue in our formulation of the stochastic system, which we present again here,

$$
\begin{aligned}
\mathrm{d}C_{ij} = &\left[r_{C_j} C_{ij} \left(1 - \left(\sum_{j=1}^{N} C_{ij} + \alpha_1 B_i \right) \right) - \gamma_{C_j} C_{ij} P_i - d_{C_j} C_{ij} \right. \\
&\left. - \beta_{C_j} C_{ij} B_i - \sigma_{C_j} C_{ij} M_i + \ a(E_j) \right] \mathrm{d}t \\
&+ \ \left[\eta_{C_j} C_{ij} + \lambda_j(t) - \eta_{BC_j} C_{ij} B_i \right] \mathrm{d}W_t, \qquad \text{(S5)} \\
\mathrm{d}B_i = &\left[r_2 B_i \left(1 - \left(B_i + \alpha_2 \sum_{j=1}^{N} C_{ij} \right) \right) - d_2 B_i \right] \mathrm{d}t + \ [\eta_2 B_i] \, \mathrm{d}W_t, \qquad \text{(S6)} \\
\mathrm{d}P_i = &\left[\sum_{j=1}^{N} \xi_j C_{ij} P_i (1 - P_i) - d_3 P_i \right] \mathrm{d}t + \ [\eta_3 P_i] \, \mathrm{d}W_t, \qquad \text{(S7)} \\
\mathrm{d}M_i = &[-d_4 M_i] \, \mathrm{d}t + \ [\eta_4 M_i] \, \mathrm{d}W_t, \qquad \text{(S8)} \\
\mathrm{d}E_j = &\left[\sum_{i=1}^{L} b C_{ij} \left(1 - \frac{E_j}{\Omega} \right) - d_5 E_j \right] \mathrm{d}t + \ [\eta_5 E_j] \, \mathrm{d}W_t, \qquad \text{(S9)}
\end{aligned}
$$

where $\lambda_j(t)$ is defined by;

$$
\lambda_j(t) = \begin{cases} 0, & \text{if } C_{ij}(t) = 0. \\ 2.873 \times 10^{-4}, & \text{otherwise.} \end{cases}
$$

and where $a(E_j)$ is defined by;

$$
a(E_j) = \begin{cases} 0.015, & \text{if } X < \frac{E_j}{\Omega} \text{ for random variable } X \sim \mathcal{U}(0,1). \\ 0, & \text{otherwise.} \end{cases}
$$

.

Having terms independent of their respective variables is necessary to ensure that the model may simulate new infections and extinction events, however this does leave the model analytically unbounded. To fix this issue, when the model was solved numerically we used min and max functions to ensure that the model was fixed between 0 and 1. To express the model in a more rigid mathematical framework, we can include the Kronecker delta function.

the Kronecker delta is defined as:

$$
\delta_{ij} = \begin{cases} 0, & \text{if } i \neq j. \\ 1, & \text{if } i = j. \end{cases}
$$

As such we can better define the model by changing equations (S5) to (S9) to,

$$\mathrm{d}C_{ij} = \Bigg[r_{C_j} C_{ij} \left(1 - \frac{\sum_{j=1}^{N} C_{ij} + \alpha_1 B_i}{K}\right) - \gamma_{C_j} C_{ij} P_i - d_{C_j} C_{ij} - \beta_{C_j} C_{ij} B_i - \sigma_{C_j} C_{ij} M_i + (1 - \delta_{(1)(C)}) a(E_j) \Bigg] \mathrm{d}t + \left[(1 - \delta_{(1)(C)})(1 - \delta_{(0)(C)})(\eta_{C_j} C_{ij} + \lambda_j(t) - \eta_{BC_j} C_{ij} B_i)\right] \mathrm{d}W_t, \tag{S10}$$

$$\mathrm{d}B_i = \left[r_2 B_i \left(1 - \frac{B_i + \alpha_2 \sum_{j=1}^{N} C_{ij}}{K}\right) - d_2 B_i \right] \mathrm{d}t + \left[(1 - \delta_{(1)(B)}) \eta_2 B_i\right] \mathrm{d}W_t, \tag{S11}$$

$$\mathrm{d}P_i = \left[\sum_{j=1}^{N} \xi_j C_{ij} (1 - P_i) - d_3 P_i \right] \mathrm{d}t + \left[(1 - \delta_{(1)(P)}) \eta_3 P_i\right] \mathrm{d}W_t, \tag{S12}$$

$$\mathrm{d}M_i = [-d_4 M_i]\, \mathrm{d}t + \left[(1 - \delta_{(1)(M)}) \eta_4 M_i\right] \mathrm{d}W_t, \tag{S13}$$

$$\mathrm{d}E_j = \left[\sum_{i=1}^{L} b C_{ij} \left(1 - \frac{E_j}{\Omega}\right) - d_5 E_j \right] \mathrm{d}t + \left[(1 - \delta_{(1)(E)}) \eta_5 E_j\right] \mathrm{d}W_t, \tag{S14}$$

where $\lambda_j(t)$ is defined by;

$$\lambda_j(t) = \begin{cases} 0, & \text{if } C_{ij}(t) = 0. \\ 2.873 \times 10^{-4}, & \text{otherwise.} \end{cases}$$

and where $a(E_j)$ is defined by;

$$a(E_j) = \begin{cases} 0.015, & \text{if } X < \frac{E_j}{\Omega} \text{ for random variable } X \sim \mathcal{U}(0, 1). \\ 0, & \text{otherwise.} \end{cases}$$

This alteration was not included in the main manuscript for the sake of readability.

S2 APPENDIX - SIMULATED ANNEALING

Inspired by the process of annealing in metallurgy, simulated annealing is a process of finding a global extrema (minimum or maximum value) of a function. The most common methods of hill-climb algorithms or gradient-descent are incredibly efficient, but for cases where there are many local extrema, these methods can become easily stuck in local extrema, and are unable to detect the “true” minimum or maximum value of the function.

Simulated annealing tackles this problem by taking a probabilistic approach. Consider a minimisation problem. Other methods will consider neighbouring points and move there if the output value is lower. Simulated annealing however, will consider a neighbouring point and instead has a *probability* of moving to this point. This probability is dependent on the value at the two points being considered, and a time-dependent variable T, that slowly decreases to 0 as the algorithm proceeds. The probability of moving to a

neighbouring position will be higher if the new position gives a desired lower value, and will also be higher the greater the value of T, i.e. the earlier it is in the process' runtime.

As time progresses, and T converges to 0, the probability of moving position also slowly converges to 0. By having the possibility of moving to a "poorer choice" of neighbour, this method searches a greater amount of the parameter space. This method was ideal for use in this project, as it was applied to minimising a cost function with a large quantity of local minima, across a very large parameter space. The cost function in our case was the distance between the model predictions and experimental data presented in Figure 2. As such the method was able to provide an accurate parameter set to use for the ensuing case studies. The drawbacks of the method are its substantially longer runtimes, which increases exponentially for multi-variate functions.
